# Entrapment of Black Carrot Anthocyanins by Ionic Gelation: Preparation, Characterization, and Application as a Natural Colorant in Yoghurt

**DOI:** 10.1021/acsomega.2c03962

**Published:** 2022-09-01

**Authors:** Melda Tavlasoglu, Gulay Ozkan, Esra Capanoglu

**Affiliations:** Department of Food Engineering, Faculty of Chemical and Metallurgical Engineering, Istanbul Technical University, Maslak, Istanbul 34469, Turkey

## Abstract

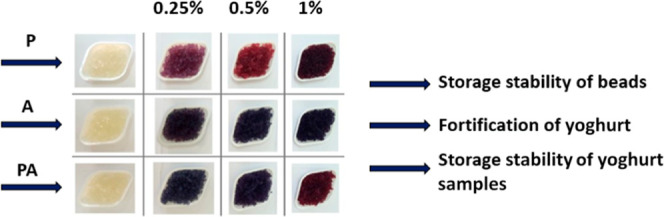

Black carrot (BC) with its potential health benefits due to the greater amount of anthocyanins and the potent antioxidant activity could be utilized as a natural colorant. The objective of this study was the entrapment of BC anthocyanins by external ionic gelation technique within the biopolymer matrix including pectin, alginate, and the mixture of both. Beads were characterized in terms of entrapment efficiency (EE), morphology, total anthocyanin content, and antioxidant capacity measured by the 2,2′-azino-bis-3-ethylbenzothiazoline-6-sulfonic acid assay. Furthermore, the color of the beads as well as yoghurt samples fortified with BC-containing beads were evaluated during storage at 4 °C for 4 weeks. While the EE for anthocyanins ranged between 47.3 and 96.6%, the antioxidant capacity changed from 50.4 to 97.7%. The maximum anthocyanin retention was found as 91.7% for 1% BC containing 1% pectin (P) + 1% alginate (A)-based beads after 4 weeks of storage. In addition, anthocyanin protection reached up to 62% and antioxidant capacity up to 55.6% in the fortified yoghurt samples containing A-based beads during storage. It is concluded that external ionic gelation could be a feasible method for BC anthocyanins due to its protective effect against acidic environment.

## 1 Introduction

There are several plants containing anthocyanins that can be used as natural food colorants.^1−5^ Black carrot (BC) (*Daucus carota* L. ssp. *sativus*) cultivation has spread to many countries, particularly Turkey and Middle and Far East.^6^ They have a bluish-purple color with high levels of anthocyanins, which have created an increasing interest to substitute the synthetic food colorants due to the legal restrictions and increasing consumer demand for natural pigments.^7^ Anthocyanins from BC show better stability against adverse conditions owing to their acylated forms with hydroxycinnamic acid and hydroxybenzoic acid as compared to other fruit or vegetable anthocyanins.^8,9^ In addition to their coloring properties, BC anthocyanins also draw attention with their health-promoting effects including the reduction of the risks of coronary heart disease, atherosclerosis, cancer, hypertension, and diabetes and prevention of inflammatory and urinary infections.^10^

Even though the interest toward natural bioactive compounds is increasing, these ingredients are susceptible to severe conditions of food processing, environmental conditions, gastrointestinal system, and other factors.^11,12^ Indeed, it has been reported that the bioaccessibility of anthocyanins was generally determined to be lower than that of other phenolic compounds,^13^ which is attributed to the structural rearrangements owing to pH changes.^14^ Therefore, microencapsulation is a promising technique to overcome such difficulties by wrapping around the active substance with a wall/carrier material.^11,15−18^

Ionic gelation is one of the microencapsulation techniques based on the ability of cross-linking polyelectrolytes in the presence of multivalent ions such as Ca^2+^, Ba^2+^, and Al^3+^ and could be carried out externally or internally.^19^ In the external gelation, Ca^2+^ ions diffuse from an external source into the polymer solution.^20^ This method has the advantages of allowing the use of heat-sensitive ingredients and promotion of gel formation without vigorous requirements.^21^ The external ionic gelation technique was successfully used by several authors.^22−24^ In these studies, entrapment efficiency (EE) depending on the type of the active compound and its characteristics,^22−24^ type and concentration of the polymer,^20^ inclusion of various additives,^4^ active/polymer ratio,^20,25^ and process variables^2^ has been analyzed. However, there is lack of information about the storage stability of beads within an acidic food matrix.

Here, we have examined, in greater detail, the effects of different polymer matrices, namely, A, P, and mixture of both (PA) on the EE of BC anthocyanins by using external gelation technique. We have also fortified the yoghurt samples with beads to investigate the stability of BC anthocyanins in an acidic environment. Moreover, anthocyanin protection ability of those bead matrices was determined during storage at 4 °C for 4 weeks.

## 2 Materials and Methods

### 2.1 Materials

Sodium azide, calcium chloride, methanol, acetic acid, and 6-hydroxy-2,5,7,8-tetramethylchroman-2-carboxylic acid (Trolox) were obtained from Sigma Chemical Co. (St. Louis. Mo. U.S.A.). The other chemicals and solvents used in this experiment were of analytical grade.

BC extract was obtained from DÖHLER—Natural Food & Beverage Ingredients (Turkey). GENU Pectin LM-101 AS was received from Kelco—Food, Beverage & Nutrition, USA, and Manugel DMB alginate was received from FMC Corporation (USA).

### 2.2 Preparation of Beads by External Ionic Gelation

The preparation of beads by external ionic gelation was adapted from Córdoba et al.^26^ P, A, and the mixture of PA stock solutions as a carrier material were prepared by dispersing powders into separate beakers at 2% (w/v) in 0.02% sodium azide containing 100 mL of deionized water. Then, the dispersions were stirred constantly at room temperature (20–25 °C) overnight for complete hydration and used for gel preparation the following day. The carrier–active material mixture was prepared by mixing the stock polymer solutions (2% P, 2% A, and 2% PA) with the BC extract at different ratios (0, 0.25, 0.5, and 1% w/v) by stirring for at least 30 min to ensure complete mixing. Then, the polymer–BC mixture was dropped into 200 mL of mildly agitated 3% (w/v) calcium chloride (CaCl_2_) solution using a 5 mL syringe with a 21 G (0.8 × 38 mm) needle. The beads were allowed to cross-link with Ca^2+^ for 30 min at room temperature and collected by filtration and washed with distilled water to remove excess Ca^2+^ on the surface. The beads were not dried to avoid any change in their properties and stored prior to usage.

### 2.3 Characterization

#### 2.3.1 Entrapment Efficiency (EE)

The EE was calculated as the ratio of bioactive mass in the beads and the mass of bioactives in the initial mixture of BC extract and coating.^27^ The EE was calculated on wet basis by eq 1.
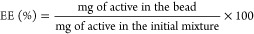
1

#### 2.3.2 Morphology

Outer structural features of beads were analyzed using an optical microscope (DS—Fi2, Nikon) with 40X magnifications.^28^

#### 2.3.3 Extraction of Anthocyanins

Prior to spectrophotometric analysis, for each sample, three independent extractions were prepared according to Ersus and Yurdagel^3^ with some modifications. 1 g of sample was treated with 25 mL of MeOH–HOAc–H_2_O (50:8:42) solvent system. The treated samples were homogenized by a homogenizer (IKA T18 Basic Ultra-Turrax) at 10,000 rpm for 30 s. Then, the samples were centrifuged (Hettich Universal 32R, Hettich Zentrifugen GmbH & Co. Tuttlingen, Germany) at 4500*g* for 20 min, and the supernatants were collected. This extraction protocol was repeated two times for the pellet, and the supernatants were pooled to a final volume of 50 mL. The prepared extracts were stored at −20 °C until analysis.

#### 2.3.4 Total Anthocyanin Content

The obtained extract for each sample was used for the determination of the total anthocyanin content by measuring the absorbance at 530 nm, which is the maximum absorbance value of anthocyanins.^29^ Anthocyanin contents are expressed as mg of cyanidin-3-*O*-glucoside equivalents per 100 g of sample.

#### 2.3.5 Total Antioxidant Capacity

Total antioxidant capacity was estimated by the 2,2′-azino-bis-3-ethylbenzothiazoline-6-sulfonic acid (ABTS) assay according to Miller and Rice-Evans.^30^ In all assays, Trolox was used as a standard, and the results were expressed in terms of mg Trolox equivalents (TE) per 100 g dry weight of the sample.

#### 2.3.6 Color Measurement

Color determinations were conducted by using a CM-3600d chromameter (Minolta Sensing Inc., Osaka, Japan). The reading of L*, a*, and b* values of samples was performed by placing the samples in Petri dishes. The readings were made in triplicate, and the equipment was calibrated with the white calibration plate before any reading.

### 2.4 Storage Stability of BC Extract-Loaded Beads

A stability study of the BC extract-containing beads was conducted in glass containers in a refrigerator at 4 °C for a period of 4 weeks. The color parameters (CieLab), the total anthocyanin content, and the total antioxidant capacities were evaluated. The aim of the storage was to investigate the effect of time on the physical and chemical properties of the BC extract-loaded beads under constant storage conditions.

### 2.5 Fortification of Yoghurt with BC-Extract-Loaded Beads

Commercial yoghurt products were purchased from local supermarkets on the day of fortification. Yoghurt formulations were made by adding 15 g of 1% BC extract-loaded beads on the basis of A, P, or PA into 50 g yoghurt samples separately and stored in glass containers in a refrigerator at 4 °C for further analysis. Physico-chemical properties were evaluated over a month at 4 °C (weekly). Plain yoghurt, 1% BC extract-containing yoghurt, and blackberry-containing commercial yoghurt were also analyzed over 4 weeks at 4 °C (weekly).

### 2.6 Statistical Analysis

Statistical analysis was applied using processed samples as independent factor versus analysis results (SPSS v. 21; SPSS Inc., Chicago, IL) by utilization of one-way analysis of variance with Tukey’s HSD post hoc test (*P* < 0.05). Error bars on figures represent standard deviations. The differences between all samples and among the samples were evaluated. Each analysis was performed in triplicate, and the results were reported as mean value ± standard deviation.

## 3 Results and Discussion

### 3.1 EE-Anthocyanin Content and Antioxidant Capacity

EE of BC anthocyanins in the beads produced by using different carrier materials are represented in Table 1. The effect of active material concentration and the type of polymer solution on EE were investigated. Results indicated that the maximum EE for anthocyanins was obtained with 0.25% BC-loaded P-based beads. Moreover, EE decreased with the increasing amount of active compound for P- and A-based beads, whereas EE increased with the increasing amount of active compound for AP-based beads.

**Table 1:** % EE of BC Anthocyanins and Antioxidant Capacity^a^

		EE	
carrier	BC extract (%)	anthocyanin content	antioxidant capacity
2% P	0.25	96.56^a^	88.16^ab^
	0.5	87.89^b^	73.04^bc^
	1	69.63^d^	65.08^cd^
2% A	0.25	71.40^cd^	88.98^ab^
	0.5	49.86^e^	50.44^d^
	1	47.28^f^	50.81^d^
1% P + 1% A	0.25	64.77^e^	97.73^a^
	0.5	70.01^cd^	79.40^abc^
	1	72.02^c^	65.48^cd^

a ^a–d^: Data presented in this table consist of average values ± standard deviation of three independent batches. Different letters in the columns represent statistically significant differences (*p* < 0.05).

Alginate-based gels possess a relatively high permeability that allows the diffusion of water and other liquids easily into the matrix which could be an advantage for immobilization of living cells and enzymes. However, it may be a drawback for bioactive protection from external factors that led to a decrease in EE when the active substance concentration enhanced from 0.25 to 1%.^31,32^ Thus, incorporation of other polymers or increasing the amount of alginate is suggested to improve the EE and the physico-chemical properties of alginate.^33−35^

While the EE of alginate-based beads was found to be 47.3% for 1% BC loading, it was improved up to 72% with the incorporation of pectin. Similarly, Pasukamonset et al.^36^ investigated the EE of *Clitoria ternatea* petal flower polyphenols through extrusion method using alginate as a coating. It was found that EE varied from 74.2 to 84.9% depending on the percentage of active compound (5–20%), alginate (1–2%), and CaCl_2_ (1.5–5%), and EE was improved by enhancing the alginate concentration. Besides, Li et al.^37^ reported the EE of two proanthocyanidin fractions obtained from *Choerospondias axillaris* fruit peels as 43.4 and 62.2%, in which EE of a fraction with higher molecular weight was found to be greater than that of a fraction with lower molecular weight. In addition to this, there were much more molecular interactions between proanthocyanidins in a fraction with higher molecular weight, indicating enhanced retention of the active substances within the gel network. In another research, the effect of fillers in order to improve the encapsulation efficiency of tea polyphenols within alginate beads was studied. According to the outcomes, EE of tea polyphenols (3 mg/mL) was found to be 38.51, 36.48, 48.56, and 57.76% for plain alginate (2%), alginate (2%) + inulin (2%), alginate (2%) + arabic gum (2%), and alginate (2%) + chitosan (1%) based beads, respectively, indicating enhanced structured network by using suitable filler substances.^38^ Flamminii et al.^39^ reported the EE of olive leaf polyphenols as 21% by incorporating into the alginate beads, which was improved to 78, 56, and 52% for the alginate–pectin, alginate–whey protein, and alginate–sodium caseinate systems, respectively.

Regarding the maintenance of antioxidant capacity after bead formation, it was found to be higher (88.2–97.7%) for 0.25% BC loading for each polymer matrix rather than others. These results are in agreement with the study of Belščak-Cvitanovic et al.,^40^ in which the recovery of antioxidant capacity of dandelion polyphenols within alginate–whey protein-based matrix produced by emulsification/internal gelation as well as alginate or pectin-based matrix obtained by external hydrophilic gelation was obtained to be more than 80%. On the other hand, the maintenance of antioxidant capacity of hibiscus extract measured by the DPPH assay was found to be in the range of 55.2–60.6% for microcapsules fabricated by ionic gelation-atomization using pectin as a carrier material, which is lower than the outcomes of the present study.^41^

### 3.2 Microscopy

**Figure 1:**
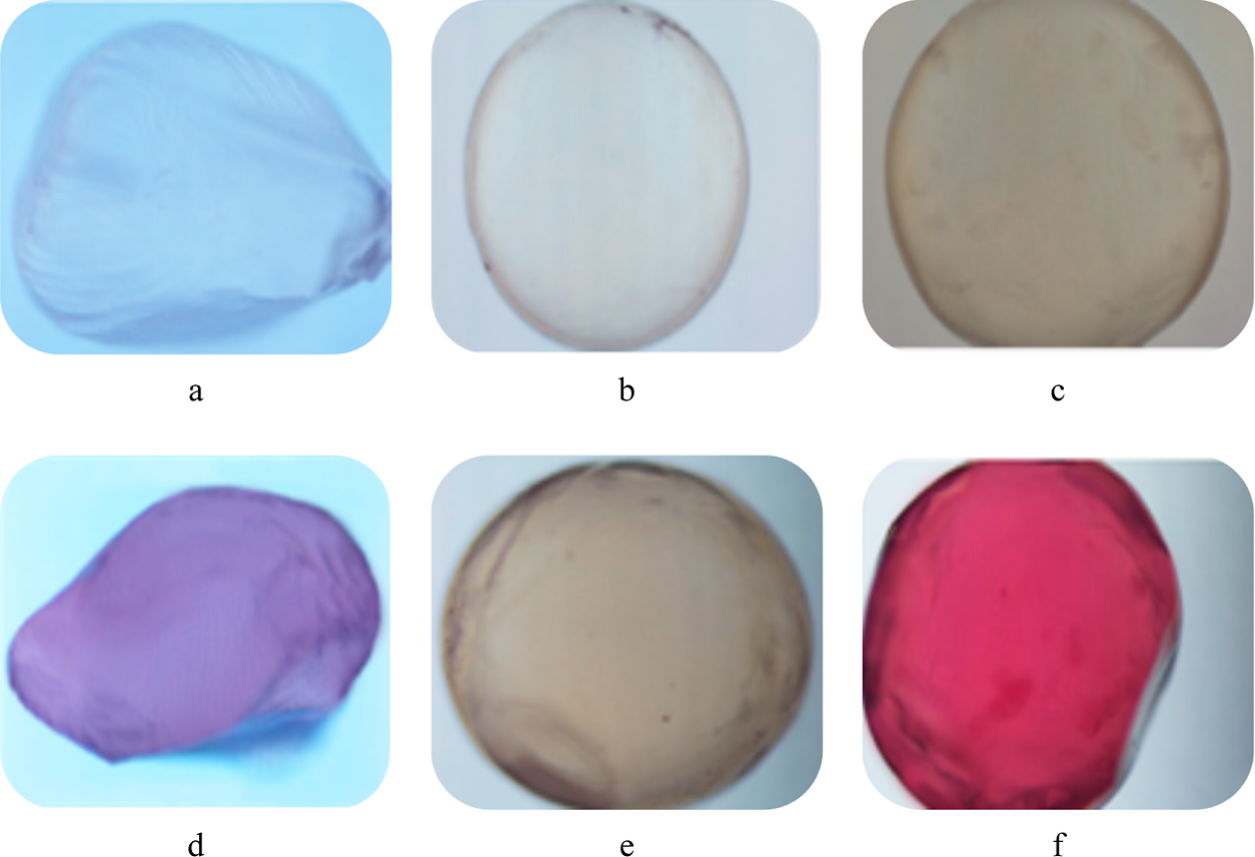
Microscopic image of (a) 2% P bead, (b) 2% A bead, (c) 1% P + 1% A bead, (d) 2% P bead + 1% BC, (e) 2% A bead + 1% BC, and (f) 1% P + 1% A bead + 1% BC.

Figure 1 shows the microstructure of beads in a light microscope at a magnification of 40X. Results indicated that beads based on A have a smoother and spherical structure among others. Similarly, Belščak-Cvitanovic et al.^41^ obtained a more rigid structure with alginate-based microcapsules, rather than pectin, chitosan, psyllium, and carrageenan-based microcapsules. Furthermore, Flamminii et al.^39^ indicated a more regular structure with alginate and alginate–pectin-based beads in comparison to alginate–whey protein and alginate–sodium caseinate-based beads. Due to the fact that the shape of the beads is one of the most important determinants, especially in complex drug-delivery systems,^42^ the beads should be tested in terms of morphology.

### 3.3 Changes in Color

CIE Lab system consists of L*, a*, and b* values in which L* is the lightness of color (100 = white, 0 = black); a* value, (+a* = red, −a* = green) and b* value, (+b* = yellow, −b* = blue). The color values of fabricated beads were also determined (Table 2). L* values showed a tendency toward decrease with the increasing BC extract concentration from 0.25 to 1%. Generally, there was a decrease in L* and b* values with higher concentration of BC extracts, while a* values increased with the additional BC content. In detail, the maximum a* value was gained with 1% BC-loaded PA-based beads.

**Table 2:** Changes in the Color Values of Beads During Storage^a^

polymer	BC extract (%)	0 week	1 week	2 weeks	3 weeks	4 weeks
2% P	0.25	L* = 7.80 ± 0.11^d^	L* = 19.90 ± 0.03^b,c^	L* = 18.05 ± 0.06^c^	L* = 22.74 ± 2.73^a,b^	L* = 23.92 ± 0.02^a^
		a* = 10.71 ± 0.04^a^	a* = 5.56 ± 0.04^b^	a* = 4.73 ± 0.10^c^	a* = 5.47 ± 0.65^b,c^	a* = 4.71 ± 0.04^c^
		b* = −3.04 ± 0.06^a^	b* = −6.72 ± 0.11^c^	b* = −5.21 ± 0.06^b^	b* = −5.01 ± 0.74^b^	b* = −4.53 ± 0.01^b^
	0.5	L* = 4.82 ± 0.12^e^	L* = 17.66 ± 0.04^c^	L* = 16.42 ± 0.01^d^	L* = 19.14 ± 0.04^b^	L* = 27.37 ± 0.03^a^
		a* = 1.71 ± 0.03^d^	a* = 18.66 ± 0.73^c^	a* = 17.95 ± 0.14^c^	a* = 21.43 ± 0.16^b^	a* = 26.00 ± 0.03^a^
		b* = 4.84 ± 0.04^c^	b* = 1.67 ± 0.06^e^	b* = 2.74 ± 0.03^d^	b* = 6.83 ± 0.11^a^	b* = 6.66 ± 0.04^b^
	1	L* = 2.86 ± 0.04^d^	L* = 9.84 ± 0.98^c^	L* = 12.76 ± 0.04^b^	L* = 13.19 ± 0.25^b^	L* = 14.06 ± 0.14^a^
		a* = 5.17 ± 0.01^b^	a* = 6.95 ± 1.00^a,b^	a* = 8.27 ± 0.06^a,b^	a* = 8.41 ± 2.97^a,b^	a* = 9.54 ± 0.10^a^
		b* = 0.38 ± 0.04^a^	b* = −2.55 ± 0.17^b^	b* = −3.37 ± 0.06^b^	b* = −3.38 ± 1.12^b^	b* = −3.03 ± 0.01^b^
2% A	0.25	L* = 11.90 ± 0.10^e^	L* = 12.69 ± 0.01^d^	L* = 25.52 ± 0.04^c^	L* = 31.60 ± 0.16^a^	L* = 26.66 ± 0.16^b^
		a* = 0.46 ± 0.04^d^	a* = 0.36 ± 0.03^d^	a* = 2.68 ± 0.10^c^	a* = 3.49 ± 0.07^b^	a* = 4.55 ± 0.11^a^
		b* = 4.12 ± 0.01^d^	b* = 2.04 ± 0.14^e^	b* = 7.42 ± 0.01^c^	b* = 10.19 ± 0.11^b^	b* = 10.81 ± 0.05^a^
	0.5	L* = 1.64 ± 0.06^e^	L* = 12.74 ± 0.06^d^	L* = 20.45 ± 0.08^c^	L* = 26.10 ± 0.37^a^	L* = 22.60 ± 0.11^b^
		a* = 3.32 ± 0.14^a^	a* = 1.12 ± 0.07^d^	a* = 1.43 ± 0.03^c^	a* = 2.78 ± 0.03^b^	a* = 2.57 ± 0.08^b^
		b* = −5.41 ± 0.03^e^	b* = −1.01 ± 0.04^d^	b* = 2.87 ± 0.08^c^	b* = 6.91 ± 0.08^a^	b* = 5.62 ± 0.02^b^
	1	L* = 0.72 ± 0.04^e^	L* = 12.58 ± 0.07^d^	L* = 14.00 ± 0.04^b^	L* = 14.19 ± 0.02^a^	L* = 13.51 ± 0.01^c^
		a* = 3.12 ± 0.04^c^	a* = 4.43 ± 0.10^a^	a* = 3.30 ± 0.07^b^	a* = 2.85 ± 0.03^d^	a* = 2.11 ± 0.04^e^
		b* = −4.43 ± 0.06^c^	b* = −8.05 ± 0.17^e^	b* = −4.83 ± 0.16^d^	b* = −2.86 ± 0.07^b^	b* = −1.29 ± 0.11^a^
1% P + 1% A	0.25	L* = 4.11 ± 0.02^d^	L* = 25.66 ± 1.24^c^	L* = 27.22 ± 0.08^b^	L* = 34.20 ± 0.04^a^	L* = 28.65 ± 0.04^b^
		a* = 1.78 ± 0.10^a^	a* = 0.62 ± 0.02^c^	a* = 0.22 ± 0.04^d^	a* = 1.00 ± 0.13^b^	a* = 1.57 ± 0.10^a^
		b* = −5.97 ± 0.04^d^	b* = 0.10 ± 0.13^c^	b* = 1.97 ± 0.07^a^	b* = 4.08 ± 0.04^a^	b* = 3.92 ± 0.15^a^
	0.5	L* = 4.69 ± 0.08^e^	L* = 22.38 ± 0.08^c^	L* = 27.26 ± 0.16^a^	L* = 25.76 ± 0.08^b^	L* = 21.89 ± 0.13^d^
		a* = 4.08 ± 0.04^a^	a* = 1.47 ± 0.10^d^	a* = 2.10 ± 0.04^c^	a* = 2.87 ± 0.09^b^	a* = 2.18 ± 0.08^c^
		b* = −5.05 ± 0.03^d^	b* = 0.78 ± 0.07^c^	b* = 4.34 ± 0.04^b^	b* = 5.67 ± 0.03^a^	b* = 4.37 ± 0.06^b^
	1	L* = 6.58 ± 0.10^d^	L* = 10.81 ± 0.04^c^	L* = 12.19 ± 0.22^a^	L* = 11.70 ± 0.11^b^	L* = 10.74 ± 0.04^c^
		a* = 28.02 ± 0.03^a^	a* = 16.87 ± 0.06^b^	a* = 15.54 ± 0.06^c^	a* = 15.75 ± 0.17^c^	a* = 10.19 ± 0.03^d^
		b* = 7.97 ± 0.09^a^	b* = −1.21 ± 0.08^b^	b* = −1.91 ± 0.06^c^	b* = −1.98 ± 0.04^c,d^	b* = – 2.16 ± 0.13^d^

a ^a–d^: Data presented in this table consist of average values ±standard deviation of three independent batches. Different letters in the rows represent statistically significant differences (*p* < 0.05).

### 3.4 Storage Stability of Beads

The effect of active material concentration and type of polymer solution on bead stability was evaluated during 4 weeks of storage under refrigeration conditions at 4 °C. The effect of storage on the total anthocyanin content of BC extract-containing beads is shown in Table 3. The results revealed that the total anthocyanin content of BC-loaded beads decreased significantly (*P* < 0.05) during 4 weeks of storage. In detail, the minimum retention (21.9%) was obtained with 0.5% BC-containing PA-based beads at the end of the storage period. On the contrary, the maximum retention was 91.7% for 1% BC-containing PA-based beads after 4 weeks of storage. The loss of total anthocyanin content of the beads at the end of storage was found to be 48.4, 36.7, and 36.1% with P-based beads, 58.8, 67.6, and 60.7% with A-based beads, and 68.1, 78.1, and 8.3% with PA-based beads for 0.25, 0.5, and 1% BC extract loading, respectively.

**Table 3:** Changes in the Total Anthocyanin Content of Beads During Storage^a^

	BC extract (%)	0 week	1 week	2 weeks	3 weeks	4 weeks
2% P	0.25	23.57 ± 1.16^a^	18.35 ± 2.12^b^	15.61 ± 0.39^c^	13.58 ± 0.7^7d^	12.16 ± 1.02^d^
	0.5	42.91 ± 1.07^a^	38.65 ± 2.22^b^	32.46 ± 0.84^c^	29.01 ± 0.61^d^	27.18 ± 2.76^d^
	1	67.99 ± 2.16^a^	57.43 ± 2.58^b^	46.26 ± 1.07^c^	44.74 ± 0.57^c,d^	43.42 ± 1.64^d^
2% A	0.25	17.43 ± 1.46^a^	9.11 ± 0.90^b^	8.60 ± 0.57^b,c^	7.79 ± 1.48^b,c^	7.18 ± 0.52^c^
	0.5	24.34 ± 1.42^a^	11.95 ± 0.90^b^	10.33 ± 0.39^c^	8.70 ± 0.69^d^	7.89 ± 0.94^d^
	1	46.16 ± 3.61^a^	33.17 ± 2.62^b^	29.41 ± 0.69^c^	21.29 ± 1.38^d^	18.15 ± 1.71^d^
1% P + 1% A	0.25	15.81 ± 1.53^a^	7.59 ± 0.41^b^	6.88 ± 0.52^b^	6.78 ± 0.51^b^	5.05 ± 1.67^c^
	0.5	34.18 ± 1.33^a^	13.27 ± 1.22^b^	8.70 ± 1.02^c^	8.20 ± 0.33^c^	7.49 ± 0.51^c^
	1	70.32 ± 1.28^a^	66.36 ± 1.89^b^	65.65 ± 0.78^b^	65.14 ± 1.83^b^	64.49 ± 0.69^b^

a ^a–d^: Data presented in this table consist of average values ± standard deviation of three independent batches. Different letters in the rows represent statistically significant differences (*p* < 0.05).

There are limited number of studies in the literature related to the storage stability of anthocyanins entrapped within beads. For instance, hibiscus anthocyanin retention within pectin (2%)-based beads obtained by dripping-extrusion method was found to be 97% after 35 days storage at 5 °C. Moreover, it has been noticed that the stability of anthocyanins may be affected by storage condition-related parameters (extrinsic) and microparticle composition-related factors (intrinsic).^2^ Li et al.^38^ studied the recovery of tea polyphenols within alginate-based hydrogel beads during storage at room temperature in the dark for 30 days. The protection ability of the bead matrices was quantified as 77.35, 80.32, 86.58, 83.65, and 85.73% for free polyphenols, alginate, alginate + inulin, alginate + gum Arabic, and alginate + chitosan systems, respectively. The results showed that the recovery of polyphenols during storage was increased with the inulin addition. In another study, the storage stability of anthocyanins within microparticles produced by dripping-extrusion method was evaluated at 5, 15, and 25 °C in the dark. The outcomes of this study indicated that anthocyanin retention was decreased by increasing the storage temperature. While maintenance of the anthocyanins was determined as 97% after 35 days at 5 °C, it decreased to 85% after 30 days at 15 °C and 26% after 20 days at 25 °C.^2^ The effect of storage on the total antioxidant capacity of BC extract-containing beads is shown in Table 4.

**Table 4:** Changes in the Total Antioxidant Capacity of Beads During Storage^a^

polymer	BC extract (%)	0 week	1 week	2 weeks	3 weeks	4 weeks
2% P	0.25	10.76 ± 3.88^a^	10.08 ± 2.36^a^	8.99 ± 1.56^a^	6.40 ± 3.78^a,b^	3.78 ± 0.31^b^
	0.5	25.02 ± 4.64^a^	24.75 ± 4.31^a^	20.00 ± 3.28^a,b^	18.64 ± 2.03^a,b^	13.20 ± 6.88^b^
	1	44.59 ± 5.22^a^	43.10 ± 4.42^a^	38.20 ± 4.04^a^	37.39 ± 5.30^a^	36.98 ± 7.00^a^
2% A	0.25	15.24 ± 1.12^a^	12.93 ± 4.75^a^	5.32 ± 2.49^b^	5.05 ± 1.92^b^	1.61 ± 0.31^b^
	0.5	17.28 ± 2.28^a^	15.65 ± 1.37^a^	10.62 ± 2.78^b^	8.85 ± 4.96^b,c^	5.59 ± 0.99^c^
	1	34.81 ± 2.45^a^	28.29 ± 2.99^a,b^	22.03 ± 3.42^b,c^	21.22 ± 5.78^b,c^	19.59 ± 2.25^c^
1% P + 1% A	0.25	16.74 ± 1.29^a^	7.5 ± 1.15^b^	7.2 ± 2.88^b^	4.87 ± 1.91^c^	2.88 ± 0.77^c^
	0.5	27.20 ± 1.43^a^	20.68 ± 5.24^b^	12.12 ± 1.72^c^	11.93 ± 4.08^c^	3.06 ± 1.91^d^
	1	44.86 ± 1.43^a^	42.28 ± 3.67^a,b^	40.65 ± 2.53^a,b^	39.70 ± 5.35^a,b^	38.07 ± 5.51^b^

a ^a–d^: Data presented in this table consist of average values ± standard deviation of three independent batches. Different letters in the rows represent statistically significant differences (*p* < 0.05).

The ABTS results achieved in this work have shown that the total antioxidant capacity of beads generally decreased significantly (*P* < 0.05) after 4 weeks of storage. While the minimum retention (10.6%) was obtained with 0.25% BC-containing A-based beads, the maximum retention was found (84.9%) for 1% BC-containing PA-based beads after 4 weeks of storage. The loss of total antioxidant capacity of the beads at the end of storage changed in the range of 17.1–75% for P-based beads, 43.7–89.4% for A-based beads, and 15.1–88.8% for PA-based beads. Our results also showed that the retention of the total antioxidant capacity was increased with the ascending concentration levels of BC extract to be encapsulated.

The current findings are in agreement with that reported by Belščak-Cvitanović et al.^41^ who found a decrease in antioxidant activity, measured by the ABTS method, of six different medicinal plants in alginate-based beads after 2 weeks of storage at 4 °C. These results may be due to the high water content of the pectin, alginate, or pectin/alginate beads. In addition to these, Li et al.^38^ noticed 77% retention ratio of tea polyphenols stored at room temperature in the dark for 30 days, whereas recovery of polyphenols changed from 80 to 86% for alginate, alginate + inulin, alginate + gum Arabic, and alginate + chitosan based bead systems. On the other hand, the antioxidant activity of alginate beads including stevia extracts, measured by ferric reducing antioxidant power and ABTS assays, did not change during the 30 days of storage at 4 °C. This result may be attributed to the increase in total phenolic content and polyphenol formation during storage.^43^

The beads obtained in this study were also evaluated in terms of their color stability during storage. Color values of beads during storage are reported in Table 2. The results showed that L* index for all bead samples tended to increase significantly (*P* < 0.05) during storage. On the other hand, there was no tendency to increase or decrease regularly regarding the a* and b* indices, but generally there was a correlation between anthocyanin retention and a* index.

### 3.5 Storage Stability of Fortified Yoghurt Samples

**Figure 2:**
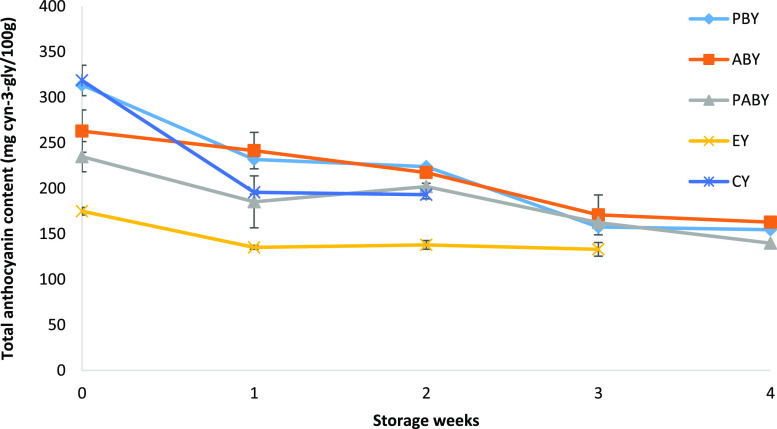
Total anthocyanin content of P bead-containing yoghurt (PBY), A bead-containing yoghurt (ABY), PA bead-containing yoghurt (PABY), extract-containing yoghurt (EY), and commercial blackberry-containing yoghurt (CY) samples stored for 4 weeks.

Yoghurt, a highly nutritional quality product, was selected to evaluate bead stability in the current pH value and acidity. The protection ability of beads was investigated by measuring the anthocyanin content, antioxidant capacity, as well as color values of the samples during 4 weeks of storage periods at 4 °C. However, plain yoghurt samples and BC extract-containing yoghurt samples after 3 weeks of storage and blackberry-containing commercial yoghurt after 2 weeks of storage could not be analyzed due to deterioration. The changes in the total anthocyanin content of all yoghurt samples are shown in Figure 2.

The results showed that the total anthocyanin content of yoghurt samples including BC-loaded beads decreased significantly (*P* < 0.05) during 4 weeks of storage. The decrease in total anthocyanin contents was found to be 50.7, 38.0, and 40.5% for P, A, and PA based beads containing yoghurt samples, respectively.

**Figure 3:**
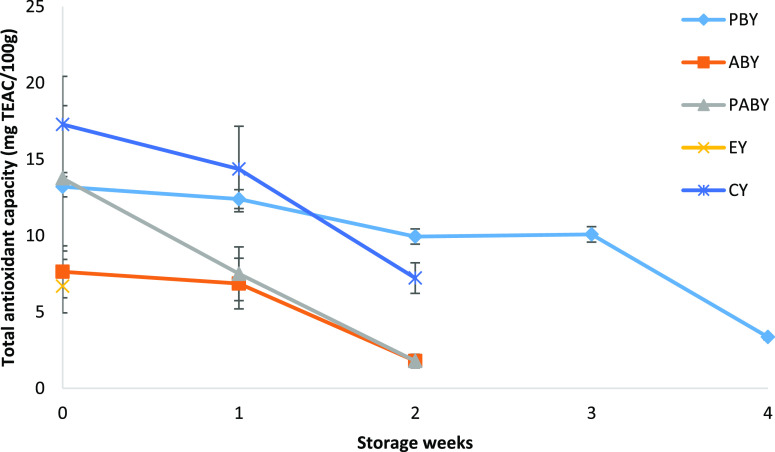
Total antioxidant capacity of P bead-containing yoghurt (PBY), A bead-containing yoghurt (ABY), PA-bead containing yoghurt (PABY), extract-containing yoghurt (EY), and commercial blackberry-containing yoghurt (CY) samples stored for 4 weeks.

The change in the total antioxidant capacity for all yoghurt samples is shown in Figure 3. The decrease in the total antioxidant capacity of commercial blackberry-containing yoghurt was obtained as 16.5% at the beginning of its deterioration, whereas there was no significant change (*P* > 0.05) in fortified yoghurt samples with beads during 3 weeks. It was clear that fortification of yoghurt by entrapped BC extract within polymer-based beads showed a protective effect on antioxidant activity against acidic conditions.

**Figure 4:**
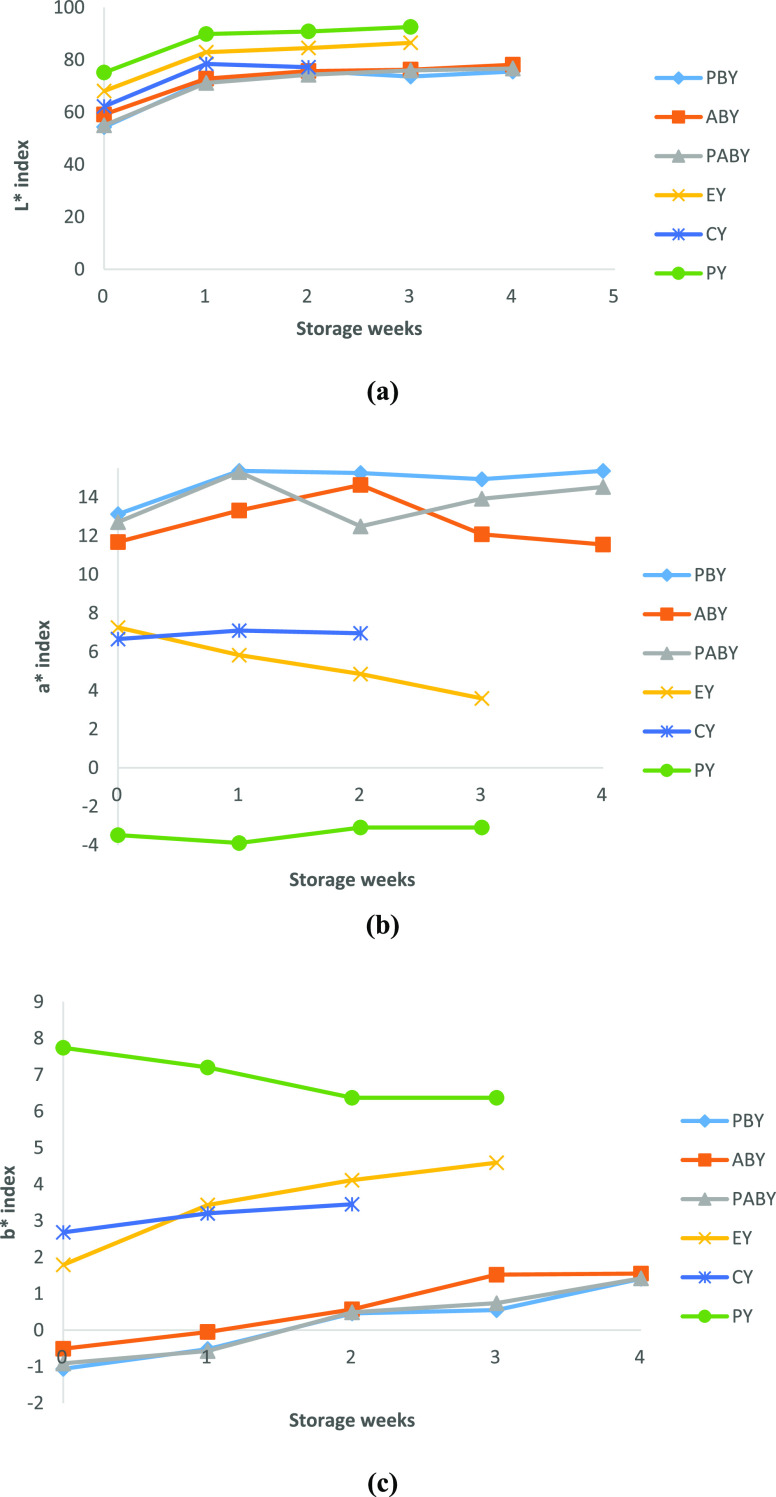
Variation of (a) L* index, (b) a* index, and (c) b* index in P bead-containing yoghurt (PBY), A bead-containing yoghurt (ABY), PA bead-containing yoghurt (PABY), extract-containing yoghurt (EY), commercial blackberry-containing yoghurt (CY), and plain yoghurt (PY) samples stored for 4 weeks.

When the extract- and BC-containing beads were added to yoghurt, they could simulate the color of blackberry-containing commercial yoghurt. Results also indicated that all colorants including beads and extract evaluated in this work had a tendency to lighten the color of the yoghurt samples during 30 days of storage (Figure 4a). However, in another study, the difference in the L* values were found to be so low for plain yoghurt, yoghurt colored by extract from red bell pepper, yoghurt with inclusion complexes, and yoghurt colored by artificial colorant stored for 60 days.^44^

The variation in the a* index for all yoghurt samples is shown in Figure 4b. Finding of this study indicated that there was no significant difference (*P* > 0.05) in a* index at the end of the storage of yoghurt samples fortified by anthocyanin-containing beads and commercial blackberry-containing samples, whereas the retention of a* value for the BC extract-containing yoghurt samples was found to be 49.5% at the end of 3 weeks.

The variation in the b* index for all yoghurt samples is shown in Figure 4c. From the outcomes of this research, it can be noticed that there was a significant difference (*P* < 0.05) during storage of all yoghurt samples except for plain yoghurt. With regard to the yoghurt samples fortified with anthocyanin-containing beads and commercial blackberry-containing samples, there was no significant difference (*P* > 0.05) between 0 and 1 week. It was highlighted that the BC-containing beads produced by external ionic gelation may be useful as a natural colorant in an acidic environment such as yoghurt.

## 4 Conclusions

In this study, pectin, alginate, and their mixture were used for the entrapment of BC extract using external ionic gelation in order to improve the functionality and stability of the bioactive compounds. It was observed that the concentration of BC and the type of the polymer affected the physico-chemical properties of beads as well as their storage stability. The EE was found to be the highest as 96.6% for 0.25% BC-loaded P-based beads. Moreover, after 4 weeks of storage, the maximum retention level for anthocyanins and the antioxidant capacity were calculated for 1% BC-containing PA-based beads as 91.7 and 84.9%, respectively. On the other hand, regarding the fortified yoghurt samples, the maximum anthocyanin recovery and antioxidant capacity were determined for the A-based bead. The results of this study showed that BC anthocyanins could be successfully entrapped in different polymer matrices including pectin, alginate, or mixture of both. These beads have the potential to be used as food colorants and food additives by incorporating into dietary supplements, functional foods, and pharmaceuticals.
